# Understanding the impact related to lifestyle interventions for people with dementia: A systematic review protocol

**DOI:** 10.1371/journal.pone.0310690

**Published:** 2024-09-26

**Authors:** Laura Middleton, Vanessa Vucea-Tirabassi, Grace Liu, Jennifer Bethell, Heather Cooke, Heather Keller, Teresa Liu-Ambrose, Megan E. O’Connell, Jackie Stapleton, Ingrid Waldron, Sarah Wu, Marie-Lee Yous, Christine Aiken, William Heibein, Myrna Norman, Carrie McAiney

**Affiliations:** 1 Schlegel-University of Waterloo Research Institute for Aging, Waterloo, Ontario, Canada; 2 School of Public Health Sciences, University of Waterloo, Waterloo, Ontario, Canada; 3 Department of Kinesiology and Health Sciences, University of Waterloo, Waterloo, Ontario, Canada; 4 Institute of Health Policy, Management and Evaluation, University of Toronto, Toronto, Ontario, Canada; 5 Toronto Rehabilitation Institute—University Health Network, Toronto, Ontario, Canada; 6 Alzheimer Society of B.C., Vancouver, British Columbia, Canada; 7 School of Nursing, University of British Columbia, Vancouver, British Columbia, Canada; 8 Department of Physical Therapy, University of British Columbia, Vancouver, British Columbia, Canada; 9 Department of Psychology and Health Studies, University of Saskatchewan, Saskatoon, Saskatchewan, Canada; 10 Library, University of Waterloo, Waterloo, Ontario, Canada; 11 Faculty of Humanities, McMaster University, Hamilton, Ontario, Canada; 12 School of Nursing, McMaster University, Hamilton, Ontario, Canada; PLOS: Public Library of Science, UNITED KINGDOM OF GREAT BRITAIN AND NORTHERN IRELAND

## Abstract

There is growing evidence to suggest that lifestyle initiatives promote brain health and reduce dementia risk. However, there is comparatively limited research focused on lifestyle interventions among people living with dementia. Most recent systematic reviews of lifestyle interventions among people living with dementia centre on the impact of exercise on cognition; yet, functional abilities and quality of life are most consistently prioritized by people living with dementia, care partners, and healthcare professionals. There is insufficient evidence to inform guidelines on effective lifestyle interventions, programs, resources, and policies for people living with dementia. To address this knowledge gap, the objective of this study is to perform a systematic review to understand the impact of lifestyle interventions among people living with dementia. The specific research questions are: "What is the effectiveness of physical activity interventions on improving functional abilities and quality of life among community-dwelling people living with dementia?", "What is the effectiveness of healthy eating/nutrition on improving nutritional status or quality of life among community-dwelling people living with dementia?" and "Does the effectiveness of interventions vary depending on the components (single or multi), setting (in-home or community centre, geography), program structure, mode of delivery, dosage, and participant characteristics (sex/gender, ethno-cultural or language group, race, dementia type)?" The results from this review will inform recommendations of lifestyle interventions and their delivery among people living with dementia in the community.

**Trial registration**: *Systematic review registration* PROSPERO #CRD42024509408.

## Introduction

The number of people living with dementia globally is expected to increase from 57.4 million people in 2019 to 152.8 million people in 2050 [1]. Dementia is currently the seventh leading cause of death and a major cause of disability and dependency among older adults [2]. Dementia is a syndrome characterized by changes in cognitive function or behaviour that are sufficient to interfere with one’s ability to function independently in daily life [3]. People living with dementia may also experience changes in physical function (including movement and balance) and sensory processing [3].

Despite medical and scientific advances, there is no cure for dementia and no treatment that can reverse dementia [4]. While emerging pharmaceutical trials offer some optimism for the treatment of Alzheimer’s disease, these treatments remain short of a cure, come with significant side effects, and do not apply to all dementia diagnoses [5]. Identification and implementation of effective non-pharmacological therapeutic strategies among people living with dementia is critically needed [6]. Although there is a growing body of evidence to suggest that modifiable lifestyle factors may reduce dementia risk and promote brain health prior to a dementia diagnosis [7], there is comparatively little research on lifestyle interventions post-diagnosis.

Physical activity and healthy eating are core parts of most public health messaging related to lifestyle to maintain health, independence, and wellbeing of older adults [8,9]. Physical activity, arguably, has the most support as an effective intervention among people living with dementia [10]. However, most exercise-related systematic reviews centre on the impact of exercise on cognition [11–17] even though there is no established minimum clinically important difference for most cognitive assessments (i.e., the change in cognitive testing that has a meaningful change in the daily function, quality of life, or lived experience of people living with dementia [18–20]. People living with dementia, care partners, and healthcare professionals identify functional abilities and quality of life most consistently as priority outcomes [21–23].

According to Alzheimer’s Disease International, healthy eating and nutrition (hereafter referred to as ‘healthy eating’) are also an important aspect of promoting health and function among people living with dementia [24]. Individuals with dementia are at an increased risk for poor food intake and declining nutritional status, and research suggests that these factors have been associated with faster cognitive and functional decline which may affect the progression of dementia after onset [25]. While there is research that dietary patterns (for example, the Mediterranean diet) may lower the risk of dementia [26–28], there is comparatively little to inform healthy eating recommendations among people living with dementia [24]. A review is necessary to understand existing evidence on interventions that can improve nutritional status and quality of life for people living with dementia and to inform eating-related guidelines research to address knowledge gaps.

There is an urgent knowledge gap related to the composition and delivery of effective lifestyle interventions on priority outcomes, which must be addressed to inform guidelines for lifestyle interventions for people living with dementia [29]. In addition, there is a need to move beyond simply supporting intervention types to understanding the most effective design and delivery of interventions, including intervention components, setting, structure, and mode of delivery [30]. These are critical unknowns to inform guidelines, programs, resources, and policy. To address this knowledge gap, a comprehensive approach to synthesizing literature on healthy lifestyle interventions for people living with dementia is needed, with specific consideration given to outcomes of interest, specifically, functional abilities and quality of life.

### Research aim

The overarching aim of this systematic review is to identify and summarize research on the effects of healthy lifestyle (i.e., physical activity, healthy eating) interventions on the functional abilities, nutritional status and quality of life of people living with dementia living in the community. A secondary aim is to identify knowledge gaps related to the effects of lifestyle interventions, paying specific attention to participants groups that may be underrepresented (e.g., by gender identity, sexual orientation, ethno-cultural group, or race). Research suggests that sex, gender, and racial differences impact the incidence of dementia, and reported rates of dementia vary widely between these groups [31]. Additionally, sex may influence the responsiveness to some interventions, but gender and race may influence the acceptability of and adherence to interventions [31,32]. Thus, it is important to use an intersectional framework to understand who is disproportionally affected by dementia and an intersectional analysis that considers sex, gender, and race.

### Research questions

The research questions are:

1. What are the effects of physical activity and healthy eating interventions (alone or in combination with each other and/or with other interventions) versus any or no control on the functional abilities, quality of life, and nutritional status (healthy eating interventions only) of community-dwelling people living with dementia?
  - What intervention characteristics (e.g., type, dose, duration), delivery characteristics (e.g., group, 1:1, independent; home, community, social context) are most effective and in whom (participant characteristics such as sex, gender identity, sexual orientation, ethno-cultural or language group, race, and dementia type)?
2. What are the knowledge gaps related to research question 1, with particular attention to underrepresented groups (ethno-cultural or language group, racial group, geography, gender identity, or sexual orientation) and knowledge needed to inform recommendations for lifestyle interventions (e.g., impacts of intervention, delivery and participant characteristics)?

## Methods

This protocol was registered within the *PROSPERO* database (CRD42024509408) and is reported in accordance with the reporting guidance provided in the Preferred Reporting Items for Systematic Reviews and Meta-Analyses Protocols (*PRISMA-P*) statement. Amendments made to this protocol with respect to conducting this systematic review will be documented and reported in both the *PROSPERO* registry and any subsequent publications.

### Eligibility criteria

#### Types of studies

Peer-reviewed intervention studies that examine the effects of physical activity and healthy eating interventions in comparison with any or no comparison group among people living with dementia will be included. These include randomized controlled trials, clinical trials, quasi-experimental designs (e.g., non-randomized, pre/post), evaluation studies, and mixed methods. To provide a comprehensive review of evidence, intervention studies with any or no comparison group (i.e., only pre-post) will be included. Studies need to include an intervention with a baseline and post-intervention outcome assessment, which may include feasibility or pilot studies. Study design will be noted and considered for subgroup analyses where possible. Observational studies (i.e., longitudinal and cohort studies, case studies) where no intervention is examined will be excluded.

#### Population

Studies conducted in any country, examining people living with dementia who are residing in the community, where participants will be individuals of any age, sex, gender, and race will be included in this review. Dementia can be determined by a documented clinical diagnosis, a self-reported clinical diagnosis, or an in-study screening process. Any type of dementia will be included (including people with major neurocognitive disorder and vascular cognitive impairment), with dementia type noted for potential subgroup analysis. We will exclude studies if the research only includes people with mild cognitive impairment or prodromal dementia. We will exclude studies conducted exclusively among people currently in acute care, transitional/convalescent care, in-patient or sub-acute rehabilitation, retirement/assisted living setting, and long-term care or nursing homes. We will exclude studies that included people living in care settings (e.g., acute, transitional or convalescent care and rehabilitation) or residing in congregate living settings (e.g., long-term care or nursing homes and senior, retirement or assisted living homes that provide assistance with activities of daily living or instrumental activities of daily living, with the exception of cleaning/housework). We will include studies with additional participant groups (e.g., care partners, people with mild cognitive impairment) and settings (e.g., long-term care, acute care) if the outcomes specific to community-dwelling people living with dementia and the place of residence are reported separately.

#### Setting

We will include studies of interventions delivered in any setting, including at home, in the community, or in health care institutions, though participants must meet the eligibility criteria described above.

### Interventions

Studies that include at least one physical activity or healthy eating intervention will be included. The interventions can be a single intervention (physical activity or healthy eating) or a multi-component intervention (physical activity and healthy eating, or either of these interventions combined with another intervention). Interventions can be individual (1 on 1) or group-based and either instructional versus independent; the mode of delivery can be in-person or virtual.

*Physical activity interventions* will include physical activity of any kind, including aerobic exercise (e.g., walking, swimming, bicycling, elliptical), flexibility training, endurance training (e.g., treadmill), strength training (e.g., resistance or weight training), balance training, and other prescribed physical activity modalities (e.g., tai chi, dance, yoga, Pilates), recreational activities (e.g., pickleball, ping pong) or sports. Rehabilitation interventions that are focused on exercise (i.e., purposeful physical activity meant to improve fitness defined by intensity and time) are eligible. All combinations of frequency, intensity, and duration of each session will be considered and noted. Interventions that do not include an active physical activity component (e.g., physical activity education, gardening) will not be considered. Rehabilitation, gait training, or technology interventions that are not focused on exercise (e.g., human robotic gait training, multi-sensory stimulation, sport stacking) will be excluded. Physical activity interventions focused solely on education or health coaching will be excluded.

*Healthy eating interventions* will include eating or nutrition interventions of any kind, including educational interventions. Possible modes of delivery can include eating or cooking groups if focused on healthy eating, nutrition education, and interventions that provide healthy foods or meals to participants. Programs focused on weight management or weight loss will be excluded. Studies solely on nutritional supplements (e.g., vitamin pills, nootropic agents, specialized drinks) will also be excluded. However, if nutrition supplements are part of the intervention (i.e., physical activity plus vitamins) then the study can be included, and the intervention will be documented.

### Types of outcome measures

We will include studies where at least one of the reported outcomes assessed is functional abilities or quality of life. For healthy eating interventions, we will also consider nutritional status as an eligible outcome.

*Functional Abilities* will include both: 1) self- and informant-reported questionnaires developed to assess the impact of functional abilities on activities of daily living and instrumental activities of daily living (e.g., Lawton-Brody Instrumental Activity of Daily Living Scale, Alzheimer’s Disease Cooperative Study–Activities of Daily Living, Functional Independence Measure). Self- versus informant-reported will be considered for subgroup analysis; and 2) performance-based assessments of functional abilities: physical performance (Short Physical Performance Battery, Senior Fitness Test), functional mobility (2- or 6-minute walk test, incident falls), strength (grip strength, chair stands), and balance (e.g., Berg Balance Scale). We will exclude outcomes related to gait and walking speed. If only cognitive outcomes are noted in the abstract (i.e., Alzheimer’s Disease Assessment Scale–Cognition), we will review the full text to determine if other relevant outcomes are included. Otherwise, articles with only cognitive outcomes will be excluded.

*Quality of Life* will include self- and informant-reported disease-specific and generic quality of life assessments. Disease-specific quality of life scales are specifically designed to capture changes in quality of life specific to dementia and changes in quality of life over the course of dementia (e.g., Dementia Quality of Life [DEMQOL], Quality of Life Alzheimer’s Disease [QOL-AD]). Generic quality of life assessments measure quality of life across a broad spectrum of disease and disability (e.g., World Health Organization Quality of Life [WHOQOL]). If a study includes both disease-specific and generic assessments, we will use the disease-specific measure for primary interpretation of intervention effects. We will record who made the quality-of-life rating (people living with dementia or informant) and consider this for subgroup analyses. We will include scales and outcomes related to overall wellbeing. However, we will exclude clinical diagnostic scales specifically related to measuring emotional or psychological symptoms (i.e., stress).

*Nutritional Status* will include self- and informant-reported assessments of nutritional status or malnutrition risk (e.g., Mini Nutritional Assessment [MNA] or Mini Nutritional Assessment-Short Form [MNA-SF], Seniors in the Community: Risk Evaluation for Eating and Nutrition [SCREEN]). We will exclude outcomes solely regarding body composition (i.e., fat mass, weight loss, body mass index), knowledge (e.g., healthy eating, recommended serving), and eating behaviours (i.e., eating patterns, food intake, preferences, usual diet). However, if the studies include body composition (i.e., body mass index) along with relevant outcomes then we will review the full text to determine if there are other relevant outcomes.

#### Exclusion criteria

Other publication types will also be excluded (e.g., review papers, protocols, letters, editorials, commentaries, opinion papers, pre-prints, conference abstracts and reviews, and grey literature).

### Search strategy

A comprehensive systematic electronic search for relevant studies will be conducted using these databases: *MEDLINE*, *EMBASE*, *Scopus* and *CINAHL*. An information specialist in consultation with other team members will develop database-specific search strategies consisting of author keywords and subject headings for each main concept: dementia, interventions (e.g., physical activity/exercise, healthy eating), outcomes (e.g., functional abilities, quality of life), and comparative studies (e.g., clinical trial). This is an iterative process, testing and modifying keywords and subject headings until a final set of search terms is complete. No date limits will be applied, although the search will be limited to English language articles. The search strategy will be based on the key components of the research question: dementia, interventions, outcomes, and comparative study. Refer to Appendix 1: Sample Search Strategies.

#### Selection of studies

Once database searches are uploaded into Covidence software, duplicate studies will be removed. The title and abstracts from each paper will be independently screened by two project team members for relevance. Any disagreements will be resolved by discussion and consensus with a third project team member brought in for decision-making (as needed). Studies which meet the inclusion criteria will be selected for full text review. Project team members will retrieve the full text papers. If the paper is not available through the University of Waterloo’s library or interlibrary loan service, the authors will be contacted. Two reviewers will independently screen each full text paper against the inclusion and exclusion to assess for relevance. Disagreement will be resolved through discussion and consensus, with a third project team member joining in the decision-making if needed. A PRISMA flow chart will be developed to show details of studies included and excluded at each stage of the study selection process.

#### Data extraction

Two reviewers will independently retrieve and record data using a data extraction form in Covidence. The form will be pilot tested across at least two studies, with modifications made to the form if needed. Any differences will be resolved by discussion, with a third investigator involved if needed. The data extracted will include: (1) identification information (e.g., title, author, year, country, geography [urban/rural/remote], organization); (2) general information (e.g., sample size, study design, allocation ratio, and study inclusion/exclusion criteria, control/comparator details); (3) participant characteristics (e.g., age, sex/gender, sexual orientation, immigrant/refugee status, ethno-cultural or language group, race, dementia type); (4) intervention component (single or multi), setting (in-home or community centre, geography), structure of program, mode of delivery (in-person versus virtual, social context), and dosage (frequency, intensity, time/duration, type); (5) reported outcome measures; and (6) results of relevant outcomes. Intervention and comparator details will align with the *Template for Intervention Description and Replication (TIDieR) Checklist* to ensure comprehensive description. Where needed, authors of papers will be contacted to request missing or additional information.

#### Quality assessment

At least two reviewers will independently assess quality and risk of bias for each study using the Risk of Bias Assessment Tool (*RoB 2*.*0*) for randomized controlled trials and the Risk of Bias In Non-randomized Studies of Interventions (*ROBIN-I*) Tool for non-randomized studies [33]. The quality assessment will be conducted within *Covidence*. Studies will be assessed to be low, unclear, or high risk of bias. Conflicts will be resolved via discussion and consensus, with a third project team member brought in if needed. Tables will be used to summarize risk of bias findings. The *GRADE* approach will be used to rate the quality of the body of evidence for each intervention/outcome pair (i.e., high, moderate, low, very low) [33]. If sufficient trials are available for a given intervention/outcome pairing (>10 studies), a funnel plot will be used to visually examine effect sizes in relation to the sample size of published studies. Asymmetry in the funnel plot indicates that publication bias is likely.

### Data synthesis and analysis

#### Descriptive analysis

Extracted information will be summarized in a tabular format. The summary table will include author, year of publication, country, study objectives, study sample descriptives (e.g., age, sex/gender, sexual orientation, immigrant/refugee status, ethno-cultural or language group, race, dementia type), details on intervention component, composition (single or multi), setting (in-home, community centre, geography), structure (instructional versus independent, group versus 1:1), mode of delivery (in-person versus virtual, social context), dose (frequency, intensity, time/duration and type), outcome measures, risk of bias, and major findings.

#### Quantitative analysis

If three of more studies describe the results of an intervention on a specific outcome (i.e., similar outcome and intervention characteristics, minimal concerns about bias), study data will be pooled using a meta-analytic approach. We plan to use a random effect model as we expect high heterogeneity across studies. *Revman5* will be used to pool estimates on outcomes of interest, which will be presented visually using forest plots. We will calculate the standardized mean difference and 95% confidence intervals for continuous outcomes (most outcomes) to pool data across different assessments [33], though we will use mean difference with 95% CI if appropriate (i.e., studies use the same outcome measures). Where available, we will use intention-to-treat data but will use completer only analyses otherwise. We will calculate between study heterogeneity using the chi-square test with I^2^ statistic to quantify unexplained heterogeneity across pooled studies. An I^2^ <25% will indicate low heterogeneity, 25% to 50% will indicate moderate heterogeneity, and over 50% will be considered high heterogeneity. Data will be pooled across studies only if I^2^ is <50% [33]. Comparisons with higher heterogeneity will be investigated using subgroup analysis [33].

#### Subgroup analysis

With sufficient data (at least three studies within relevant subgroups), we will conduct subgroup analysis based on intervention characteristics, including intervention component, composition (single or multi), setting (in-home, community centre, geography), structure (instructional versus independent, group versus 1:1), mode of delivery (in-person versus virtual, social context), dose (frequency, intensity, time/duration and type), and outcomes in each subgroup. We will also stratify based on participant characteristics (sex/gender, ethno-cultural or language group, race, dementia type), study design, high versus low risk of bias, self- versus informant-reported outcomes, and adherence over versus under 80%.

## Discussion

The purpose of this review is to summarize the effects of physical activity and healthy eating interventions on functional abilities, quality of life, and nutritional status (healthy eating interventions only) of people living with dementia. By focusing on these outcomes we will better reflect the priority outcomes identified by people living with dementia and their care partners [21–23]. Exploring the effectiveness by intervention dose and delivery will also allow us to better inform guidelines regarding interventions, programs, and policy related to lifestyle interventions, The review will also help to identify key gaps in the current literature and thereby inform future research.

Research on the identification and implementation of non-pharmacological therapeutic strategies for people living with dementia is critically needed [6,34,35]. The results from this knowledge synthesis will highlight what is known, and not known, about the impacts of physical activity and healthy eating interventions for people living with dementia in the community. Importantly, we will investigate the effectiveness of single and multi-component interventions in relation to setting, structure of program, mode of delivery, and dosage. We will also conduct subgroup analysis regarding participant characteristics if the data are adequately reported in the included studies.

Most prior reviews have focused on a single intervention (most often, exercise) on few outcomes (most often, cognition) [11–17]. While a recent review attempted to identify the most appropriate physical activity interventions by type and dose for improving both cognitive function and functional abilities [36], eligibility was restricted randomized controlled trials. Our review’s broad inclusion criteria will ensure both a comparatively larger sample size and broader scope of interventions (i.e., exercise and/or heathy eating, single or multi-component), which will give us a more holistic understanding of these interventions, including the impacts of specific intervention characteristics. For example, we expect that interventions that integrate social connections to be more effective than home-based interventions. Social engagement and supports are a strong predictor of quality of life among people living with dementia [37]. Past reviews have not considered the social context of interventions as a moderator of effects.

We anticipate that the results of this review will provide guidance for healthcare professionals, policymakers, and researchers to design and implement policies and interventions for physical activity and healthy eating with the goal of improving the health and wellbeing of people living with dementia living in the community.

## Supporting information

S1 File (DOCX)

S2 File (DOCX)
