# Supplementary material for: Understanding the impact related to lifestyle interventions for people with dementia: A systematic review protocol
Source: PLoS One. 2024 Sep 26;19(9):e0310690. doi: 10.1371/journal.pone.0310690 (PMC11426514; doi:10.1371/journal.pone.0310690)
Supplement: S1 File — (DOCX) [file pone.0310690.s001.docx]

**Supporting Information**

**Appendix 1: Sample Search Strategies (MEDLINE and EMBASE – OVID Platform)**

*Comparative study*

**MEDLINE:** exp controlled study/ or exp clinical trial/ or intervention study/ or clinical study/ or (intervention stud* or intervention group* or (pre adj3 post) or pretest or posttest or experimental stud* or quasi-experimental or nonrandom* or non random* or random* or clinical study or clinical trial or control* trial or control* study or comparative stud* or (control* adj3 group)).ti,ab,kf. or

((pre or post) adj1 (intervention* or program* or test*)).ti,ab,kf.

not (review or systematic review or meta-analysis or letter or comment or case reports or editorial).pt.

**EMBASE:** (intervention stud* or intervention group* or (pre adj3 post) or pretest or posttest or quasi-experiment* or experimental stud* or nonrandom* or non random* or random* or clinical stud* or control* stud* or (control* adj3 group) or ((pre or post) adj1 (intervention* or test* or program*)) or trial* or comparative stud*).mp. or "Controlled Before-After Studies"/ or exp clinical trial/ or case control studies/ or evaluation study/

not (conference abstract or conference review or letter or note or preprint or review).pt.

*Persons with dementia*

**MEDLINE:** (dementia or alzheimer* or Arteriosclerotic encephalopath* or Binswanger* encephalopath* or Binswanger* disease or Subcortical leukoencephalopath* or (Subcortical adj3 encephalopath*) or pick* disease or primary progressive aphasia or frontotemporal lobar degeneration or hereditary dysphasic disinhibition or Wilhelmsen-lynch or lewy body disease).ti,ab,kw. or exp dementia/ or exp alzheimer disease/

not (exp animals/ not humans/)

**EMBASE:** (dementia or alzheimer* or Arteriosclerotic encephalopath* or Binswanger* encephalopath* or Binswanger* disease or Subcortical leukoencephalopath* or (Subcortical adj3 encephalopath*) or pick* disease or primary progressive aphasia or frontotemporal lobar degeneration or hereditary dysphasic disinhibition or Wilhelmsen-lynch or lewy body disease).ti,ab,kf. or exp dementia/

not (exp animals/ not humans/ or exp experimental animal/ or exp animal experiment/ or exp animal model/ or exp rodent/ or (rat or rats or rodent or rodents or animal or mice or mouse).ti.)

*Physical activity interventions*

**MEDLINE:** (Physical activit* or Exercis* or Aerobic* or Walking or walk or Dance or dancing or Yoga or Tai chi or Taichi or Treadmill or Cycling or Swim* or aquatic or physical training or physical conditioning or fitness or bicycling or elliptical or running or recreational activit* or leisure activit* or ((Strength or resistance or balance or multicomponent or multimodal or multidomain or weight or endurance) adj1 (training or intervention or program*))).ti,ab,kw. or exp exercise/ or exp exercise therapy/ or exp exercise movement techniques/ or sports/ or bicycling/ or running/ or jogging/ or walking/ or swimming/

**EMBASE:** (Physical activit* or Exercis* or Aerobic* or Walking or walk or Dance or dancing or Yoga or Tai chi or Taichi or Treadmill or Cycling or Swim* or aquatic or physical training or physical conditioning or fitness or bicycling or elliptical or running or recreational activit* or leisure activit* or ((Strength or resistance or balance or multicomponent or multimodal or multidomain or weight or endurance) adj1 (training or intervention or program*))).ti,ab,kf. or exp exercise/ or exp physical activity/ or kinesiotherapy/ or dynamic exercise/ or leg exercise/ or movement therapy/ or muscle training/ or pilates/ or stretching exercise/ or tai chi/ or exp yoga/

*Healthy eating interventions*

**MEDLINE:** (nutritionist* or dietician* or dietitian* or cooking).ti,ab,kw. or nutritionists/ or diet therapy/ or diet therapy.fs. or nutrition therapy/ or

((intervention* or program* or guidance or education* or coach* or advice or advise or counsel*).ti,ab,kw. or education.fs. or health education/ or health promotion/ or patient education as topic/) and ((healthy eating or food or nutrition* or diet or dietary or meal or meals or mealtime* or fruit* or vegetable* or nuts or lifestyle).ti,ab,kw.)

**EMBASE:** (nutritionist* or dietician* or dietitian* or cooking).ti,ab,kf. or Dietitian/ or Diet therapy/ or Nutrition education/ or nutritional counseling/ or

(healthy eating or food or nutrition* or diet or dietary or meal or meals or mealtime* or fruit* or vegetable* or nuts or lifestyle).ti,ab,kf. and ((intervention* or program* or guidance or education* or coach* or advice or advise or strateg* or counsel*).ti,ab,kf. or therapy.fs.) (nutritionist* or dietician* or dietitian* or cooking).ti,ab,kw. or nutritionists/ or diet therapy/ or diet therapy.fs. or nutrition therapy/

*Outcomes*

**MEDLINE:** (functional performance or functional abilit* or functional status or functional capacity or functional dependence or muscle or grip strength or hand strength or leg strength or handgrip or hand grip or mobility or gait or balance or fall or falls or flexibil* or walk* test* or walk* speed* or chair stand* or isometric strength or "timed up and go" or activities of daily living or iadl or quality of life or independence or well being or wellbeing or physical performance or fitness or exercise test* or nutrition assessment or malnutrition or mini nutritional assessment or nutritional status or MNA).ti,ab,kw. or range of motion, articular/ or exp postural balance/ or exp muscle strength/ or exp physical fitness/ or exp gait/ or accidental falls/ or activities of daily living/ or independent living/ or self care/ or functional status/ or exp quality of life/ or psychological well-being/ or exp exercise test/ or exp physical endurance/ or malnutrition/

**EMBASE:** (functional performance or functional abilit* or functional status or functional capacity or functional dependence or muscle or grip strength or hand strength or leg strength or handgrip or mobility or gait or balance or fall or falls or flexibil* or walk* test* or walk* speed or chair stand* or isometric strength or "timed up and go" or activities of daily living or iadl or quality of life or independence or well being or wellbeing or physical performance or fitness or exercise test* or nutrition assessment or malnutrition or mini nutritional assessment or nutritional status or MNA).ti,ab,kf. or agility/ or body movement/ or exp limb movement/ or physical mobility/ or range of motion/ or exp body equilibrium/ or exp "muscle characteristics and functions"/ or exp hand strength/ or fitness/ or functional status/ or functional status/ or gait/ or falling/ or daily life activity/ or independent living/ or independence/ or self care/ or exp quality of life/ or wellbeing/ or physical well-being/ or emotional well-being/ or psychological well-being/ or frailty/ or physical endurance/ or physical performance/ or physical capacity/ or malnutrition/

*Limits:*

**MEDLINE and EMBASE**: limit to English language
